# Analysis of prognostic factors for survival in patients with primary spinal chordoma using the SEER Registry from 1973 to 2014

**DOI:** 10.1186/s13018-018-0784-3

**Published:** 2018-04-06

**Authors:** Yue Pan, Lingyun Lu, Junquan Chen, Yong Zhong, Zhehao Dai

**Affiliations:** 1 Department of Spine Surgery, the Second Xiangya Hospital, Central South University, Changsha, 410011 China; 2 Department of orthopaedics, the Fifth Hospital of Xiamen, Xiamen, 361101 China; 3 Department of Nephrology, Xiangya Hospital of Central South University, Changsha, 410008 China

**Keywords:** SEER, Spinal chordoma, Prognostic factors, Osseous neoplasm

## Abstract

**Background:**

Spinal chordomas are rare primary osseous tumors that arise from the remnants of the notochord. They are commonly considered slow-growing, locally invasive neoplasms with little tendency to metastasize, but the high recurrent rate of spinal chordomas may seriously affect the survival rate and quality of life of patients. The aim of the study is to describe the epidemiological data and determine the prognostic factors for decreased survival in patients with primary spinal chordoma.

**Methods:**

The Surveillance, Epidemiology, and End Results (SEER) Registry database, a US population-based cancer registry database, was used to identify all patients diagnosed with primary spinal chordoma from 1973 to 2014. We utilized Kaplan–Meier method and Cox proportional hazards regression analysis to evaluate the association between patients overall survival and relevant characteristics, including age, gender, race, disease stage, treatment methods, primary tumor site, marital status, and urban county background.

**Results:**

In the data set between 1973 and 2014, a total of 808 patients were identified with primary spinal chordoma. The overall rate of distant metastatic cases in our cohort was only 7.7%. Spinal chordoma was more common occurred in men (62.6%) than women (37.3%). Majority of neoplasms were found in the White (87.9%), while the incidence of the Black is relatively infrequent (3.3%). Three hundred fifty-seven spinal chordomas (44.2%) were located in the vertebral column, while 451 patients’ tumor (55.8%) was located in the sacrum or pelvis. Age ≥ 60 years (HR = 2.72; 95%CI, 1.71 to 2.89), distant metastasis (HR = 2.16; 95%CI, 1.54 to 3.02), and non-surgical therapy (HR = 2.14; 95%CI, 1.72 to 2.69) were independent risk factors for survival reduction in analysis. Survival did not significantly differ as a factor of tumor site (vertebrae vs sacrum/pelvis) for primary spinal chordoma (HR = 0.93, *P* = 0.16). Race (*P* = 0.52), gender (*P* = 0.11), marital status (*P* = 0.94), and urban background (*P* = 0.72) were not main factors which affected overall survival rate.

**Conclusion:**

There was no significant difference in overall survival rate between chordomas located in the sacrum and vertebral column. Spinal chordoma patients with an elderly age (age ≥ 60), performing non-surgical therapy, and distant metastasis were associated with worse overall survival. Performing surgery was an effective and reliable treatment method for patients with spinal chordoma, and public health efforts should pay more attention to the elderly patients with spinal chordoma prior to distant metastasis.

## Background

Chordoma, a rare primary bone tumor which arose from the remnants of the notochord and occurred along the spinal axis from the clivus to the sacrum, typically affected those in the 40- to 60-year-old age group but had been reported in children and the very elderly [1–3]. The annual incidence rate of new diagnoses of chordoma was approximately one to two cases per million each year across countries with a slightly higher frequency in male than female. Chordoma was a low-grade, slow-growing but locally invasive and aggressive tumor. The adjacent muscular, nervous tissue, and related joints were infiltrated gradually. The distant metastases were rarely reported and only occurred late in the disease, but high recurrent rate seriously affected the survival rate and quality of life of patients, and the overall 5-year survival was approximately 50% only [4, 5]. More than 60% of chordoma were located on the sacrum and vertebral bodies, and abundant studies demonstrated that spinal chordoma owns longer mean survival rate than other types of primary spinal tumor [6–9], but the survival and prognosis factors of spinal chordoma were not well described. What is more, the epidemiological data of spinal chordoma was controversial and scarce.

The Surveillance, Epidemiology, and End Results registry (SEER) Program, maintained by the National Cancer Institute (NCI), is the only comprehensive source of population-based information in the USA that collects cancer-related survival data on cancer cases from 1973. The SEER Program covers approximately 28% of the US population [10], thus encompassing a much larger sample size than would otherwise be possible with any single institution and most multi-institutional experiences. In this study, the Cox proportional hazards regression was used to analyze the association between patients overall survival and relevant characteristics, including age, gender, race, disease stage, treatment methods, primary tumor site, marital status, and urban background based on the SEER database. Thus, the goal of the present study was to conduct a large population-based study to describe the epidemiological data of patients with chordomas, evaluate the prognostic factors for decreased survival and establish standard treatment strategy.

## Methods

### Study criteria

Patients in SEER Registry diagnosed with chordoma from 1973 to 2014 were identified using the Histologic International Classification of Disease for Oncology, 3rd Edition (IDO-O-3 code 9370). And then, the primary tumor which was located on the vertebral column (code 412 in ICD-O-3) or sacrum/pelvis (code 414 in ICD-O-3) in chordoma patients was analyzed. Information on 808 spinal chordoma patients was available in the database, including patient demographics, primary tumor site, disease stage, treatment methods, marital status, urban background, and follow-up information. Patient’s age was recorded in the SEER database as a categorical variable in 5-year intervals, beginning at 0 years and ending at 85 years or more. Based on the previous studies [6, 11–14], we supposed that the outcomes and clinical features may be different between children and adolescence, younger adult patients, and older patients, so we assigned patients into three groups (0- to 20-year-old age group, 21- to 59-year-old age group, and over 60-year-old age group). In addition, we classified patients coded with “distant” as metastatic, while patients coded with “localized” or “regional” as non-metastatic. Patients were censored when a patient was alive at the time of last follow-up. Primary outcome was defined as time in months from diagnosis to death from any cause for overall survival.

The raw data in this study was downloaded from SEER web site (https://seer.cancer.gov/data/) via SEER*Stat in client-server mode after we submitted a request for access and signed the SEER research data agreement.

### Statistical analysis

SPSS (version 17.0.1; Chicago, IL, USA) was used for statistical analysis. The Kaplan–Meier method was used to calculate the overall survival rate. The log-rank test was used to formally test the differences. A probability value < 0.01 was considered statistically significant. Multivariate analyses were performed using Cox proportional hazard ratios in order to identify independent predictors of survival. Hazard ratio (HF) > 1 revealed that prognostic factor is associated with decreasing in survival, while HR < 1 revealed that prognostic factor is associated with increasing in survival rate compared with the reference. HR = 1 indicated that there was no significant relationship between them.

## Result

**Fig. 1:**
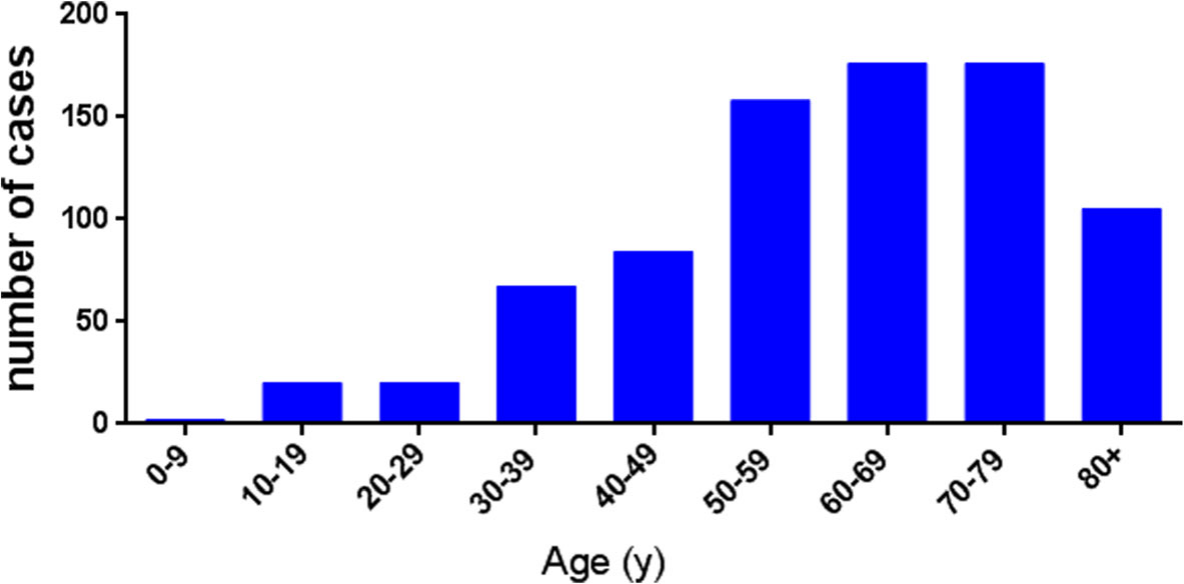
Number of spinal chordoma cases according to age at diagnosis from 1973 to 2014 in SEER

**Fig. 2:**
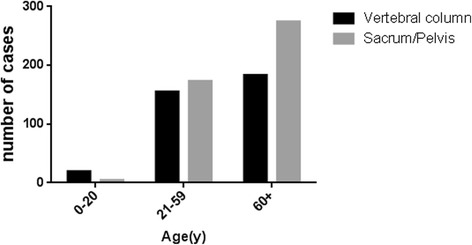
The distribution of primary site of spinal chordoma according to three different age groups (0- to 20-year-old age group, 21- to 59-year-old age group, over 60-year-old age group)

In the data set from 1973 to 2014, a total of 808 primary spinal chordoma patients was identified with a significantly higher frequency in male (62.6%) than in female (37.4%) (Table 1). Majority (87.9%) of neoplasms were found in the white, while 3.3% of neoplasms were found in the black. The majority of cases were seen in people older than 30 years old (94.8%), and the frequency of spinal chordoma increased with age (Fig. 1). The distribution of primary site also varied with age (Fig. 2). Overall, 357 spinal chordomas (44.2%) were located in the vertebral column, and 451 patient tumors (55.8%) were located in the sacrum or pelvis, respectively. The overall rate of distant metastatic cases in our cohort was 7.7%, and majority of spinal chordoma patients (67.9%) received surgical treatment.

**Table 1:** Characteristics of 808 patients with primary spinal chordoma registered in the SEER database (1973–2014)

Category	No. (%)
Age in years	
0–20	21 (2.6)
21–59	330 (40.8)
60+	457 (56.6)
Gender	
Male	506(62.6)
Female	302 (37.4)
Race	
White	711 (87.9)
Black	26 (3.3)
Other	71 (8.8)
Primary site	
Vertebrae	357 (44.2)
Sacrum/pelvis	451 (55.8)
Disease stage	
Localized/regional	654 (80.9)
Distant	62 (7.7)
Unstaged	92 (11.4)
Therapy performed	
Surgical	549 (67.9)
Non-surgical	259 (32.1)
Marital status	
Unmarried	132 (16.3)
Married	676(83.7)
Rural or urban	
Rural	141 (17.5)
Urban	667 (82.5)

Overall survival and disease-specific survival were 80.5 and 89.0% at 3 years, 68.4 and 80.9% at 5 years, and 39.2 and 60.1% at 10 years. The median overall survival and disease-specific survival was 7.8 years and 13.8 years respectively. Analysis of Kaplan–Meier survival curves with the log-rank test revealed that non-surgical therapy (*p* < 0.01), distant metastasis (*p* < 0.01) and age ≥ 60 years old (*p* < 0.01) were associated with the decreased overall survival (Table 2). The following parameters had no association with the overall survival in univariate analysis: race, gender, urban county background, and marital status.

**Table 2:** Univariate analysis of the prognostic factors of primary spinal chordoma patients

Variable	5-year overall survival (%)	Median overall survival (months)	*p* value
Age in years			< 0.001
0–20	65.5	138	
21–59	84.4	146	
60+	56.1	69	
Gender			0.192
Female	71.1	99	
Male	65.8	89	
Race			0.061
White	59.0	91	
Black	67.3	114	
Other	68.1	85	
Primary site			0.992
Vertebrae	68.0	95	
Sacrum/pelvis	68.5	92	
Disease stage			< 0.001
Localized/regional	69.6	102	
Distant	51.6	80	
Unstaged	61.7	86	
Therapy performed			< 0.001
Non-surgical	48.2	56	
Surgical	74.7	111	
Marital status			0.454
Unmarried	69.5	98	
Married	68.0	91	
Rural or urban			0.326
Rural	64.4	91	
Urban	69.5	95	

Calculated using log-rank test, *p* < 0.01 considered statistically significant

Multivariate models were created in order to identify independent predictors in spinal chordoma patients, and cox proportionate hazard ratios were evaluated for data (Table 3). Age ≥ 60 years old (HR = 2.72; 95%CI, 1.71 to 2.89), distant metastasis (HR = 2.16; 95%CI, 1.54 to 3.02), non-surgical therapy (HR = 2.14; 95%CI, 1.72 to 2.69) were independent risk factors for survival in models. Survival did not significantly differ as a factor of tumor site (vertebrae vs sacrum/pelvis) for primary spinal chordoma (HR = 0.93, *P* = 0.16). Race (*P* = 0.52), gender (*P* = 0.11), marital status (*P* = 0.94), and urban background (*P* = 0.72) were not main factors which affected overall survival rate.

**Table 3:** Multivariate analysis of patients with primary spinal chordoma identified in the SEER Program database from 1973 to 2014

Variable	HR (95%CI)	*P* value
Age in years		< 0.001
0–20	Ref	
21–59	1.04 (0.65–1.10)	
> 60	2.72 (1.71–2.89)	
Gender		0.11
Female	Ref	
Male	1.18 (0.96–1.44)	
Race		0.52
White	Ref	
Black	0.48 (0.23–1.03)	
Other	0.89 (0.61–1.30)	
Primary site		0.16
Vertebrae	Ref	
Sacrum/pelvis	0.87 (0.71–1.06)	
Disease stage		< 0.001
Localized/regional	Ref	
Distant	2.16 (1.54–3.02)	
Unstaged	1.01(0.75–1.34)	
Rural or urban		0.72
Urban	Ref	
Rural	0.96 (0.77–1.21)	
Therapy performed		< 0.001
Surgical	Ref	
Non-surgical	2.14 (1.72–2.69)	
Marital status		0.94
Unmarried	Ref	
Married	0.99 (0.76–1.29)	

Calculated using Cox proportional hazards model and the values are given as the odds ratio, with the 95% confidence interval in parentheses

*HR* indicates hazard ratio, *CI* indicates confidence interval, *Ref* indicates reference

## Discussion

**Fig. 3:**
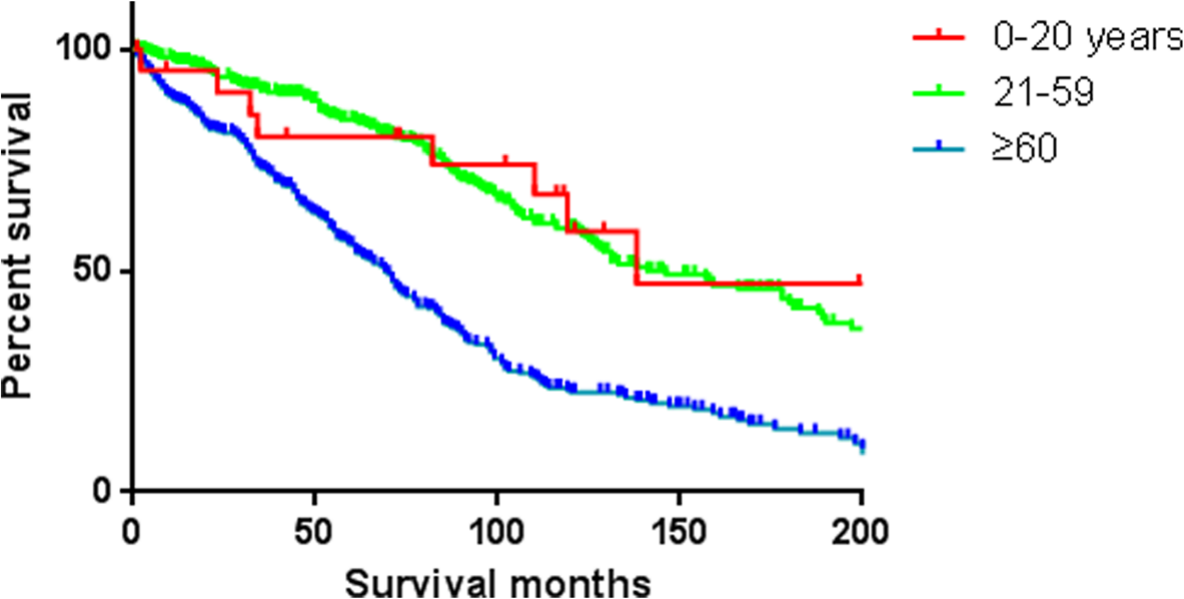
Kaplan–Meier estimated overall survival in spinal chordoma patients, stratified by different diagnostic age groups (0- to 20-year-old age group vs 21- to 59-year-old age group vs over 60-year-old age group)

**Fig. 4:**
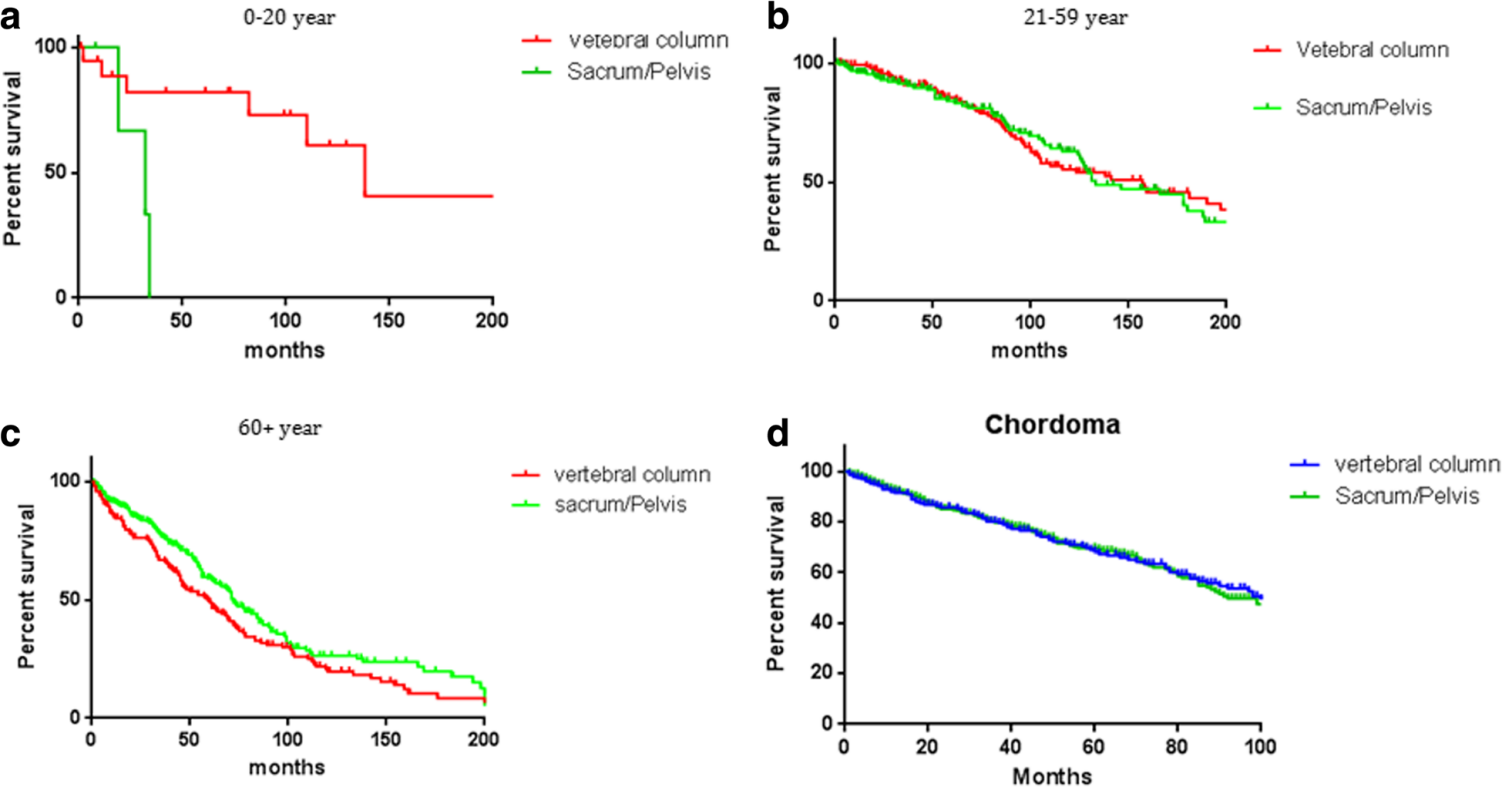
Kaplan–Meier estimated overall survival which is stratified by primary site (vertebral column vs sacrum/pelvis) in spinal chordoma patients from different ages (**a** 0- to 20-year-old age group, **b** 21- to 59-year-old age group, **c** over 60-year-old age group, and **d** all age groups)

In this study, we provided a population-based survival estimate for patients with primary spinal chordoma. Previous studies reported that chordoma typically affected those in the 40- to 60-year-old age group, and age older than 59 years was an independent predictor for reducing survival rate in chordoma patients [6, 11–13]. In our study, the majority of spinal chordoma cases were identified in people over the age of 30 years old, and most of these cases occurred in people over 60 years old (Fig. 1). Compared with the 0- to 20-year-old age group and 21- to 59-year-old age group, the patients in the over 60-year-old group had a poor prognosis, and aging ≥ 60 years old was identified as an independent risk factor for survival. The poor prognosis in older patients may be because of the similar symptoms of spinal chordoma at an early stage and other benign bone pathologies, such as lumbar muscle strain, degenerative lumbar disc disease, and spinal stenosis that confound patients and their physicians and may lead to a delayed diagnosis. There was no statistically significant difference between 0- and 20-year-old age group and 21- and 59 year-old age group in overall survival (Fig. 3). What was worth mentioning was that the median survival of the only four patients with sacrum chordoma in the 0- to 20-year-old age group in SEER was only 32 months, while the median survival of vertebral chordoma in the same group was 138 months (Fig. 4). Coffin et al. [14] reported a study included 12 children (mean age of 6 years) with chordoma, where 10 of them had died at intervals of 3 weeks to 4.5 years after diagnosis. They argued that chordomas in children were more variable histologically and may pursue a more aggressive clinical course than those in adults. These implied that the tumor site of chordoma in children and adolescents may be an independent factor for the survival rate, which needed to be confirmed further by more patient data. However, no significant difference in survival rate was found between sacrum chordoma and mobile spinal chordoma in patients over the age of 20 years old (21- to 59-year-old age group, *P* = 0.23; over 60-year-old age group, *P* = 0.02) and overall survival rate did not differ by primary tumor site significantly (Fig. 4).

**Fig. 5:**
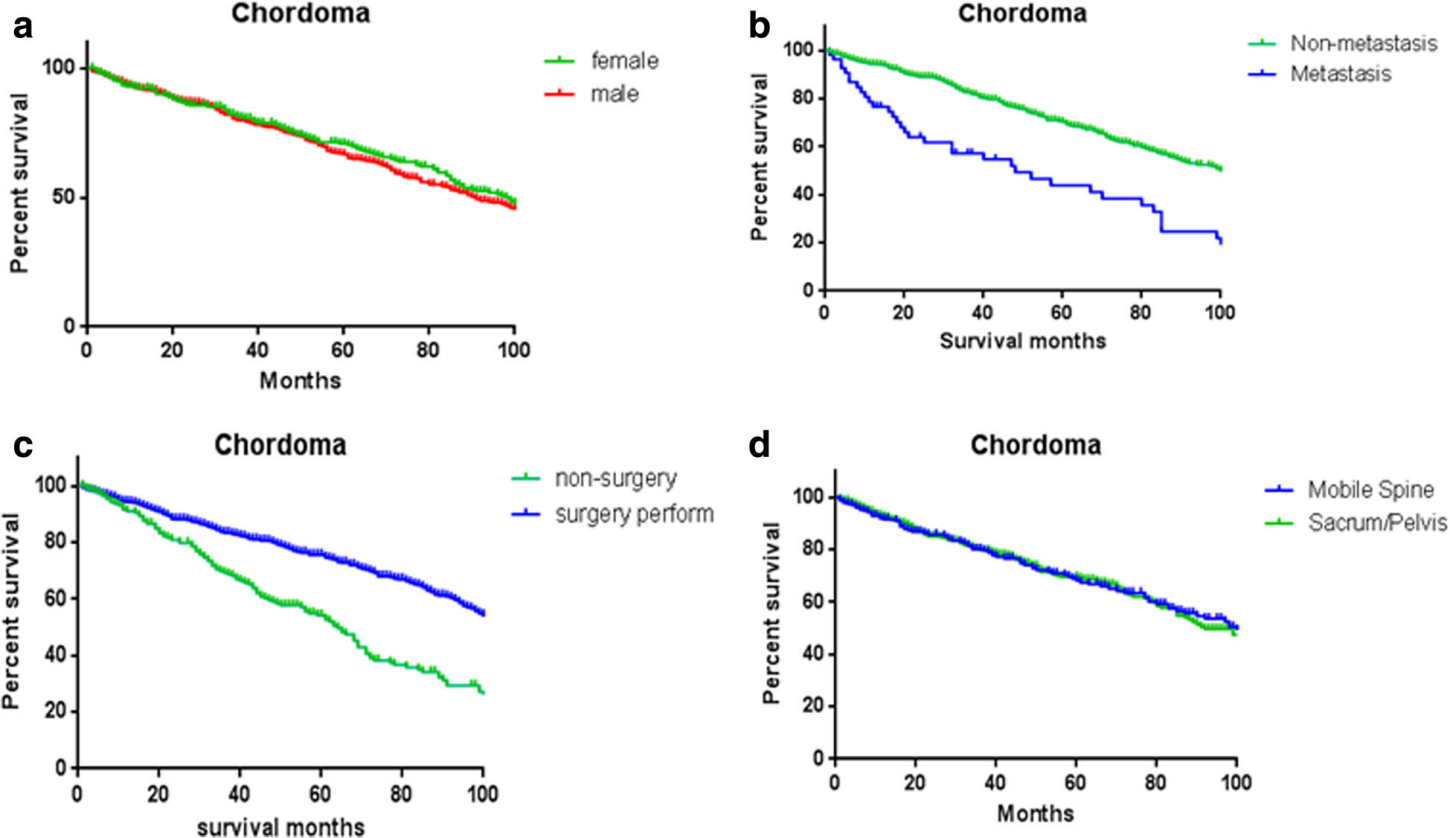
Kaplan–Meier estimated overall survival in all spinal chordoma patients, stratified by **a** gender (female vs male), **b** metastasis status at presentation (non-metastasis vs metastasis), **c** therapy method (no surgery vs surgery perform), and **d** primary site (vertebral column vs sacrum/pelvis)

On the other hand, spinal chordomas were more common occurred in men (62.6%) than in women (37.4%) and there was no significant difference in overall survival rate between men and women in our study (Fig. 5). Furthermore, gender was found not to be an independent factor which affected the survival rates after multivariate cox analysis of SEER data. Previous studies reported that the location distribution of chordomas was 50–60% sacral, 25–30% spheno-occipital, and 15% occurred in the vertebral bodies of the mobile spine (most commonly the C2 vertebrae followed by the lumbar and then thoracic spine) [15–17]. Similar to our results, 357 spinal chordomas (44.2%) were located in the vertebral column, and 451 patients’ chordomas (55.8%) were located in the sacrum or pelvis. Upon further investigation, we found that the distribution of primary site varied with age. Majority of young patients’ chordomas were found in the vertebral column, while a great amount of old patients’ chordomas was found in sacrum or pelvic (Fig. 2).

It was no doubt that surgery was the effective and reliable treatment method for all spinal lesion [18], including chordoma, although the risk for operation was high and some patients showed worse life qualities after surgery including postoperative pain, anxiety and functional deficits when sacrificing adjacent neurovascular structures in en bloc sacrum resection [19]. In our analyses, the majority of the cases underwent surgical treatment (67.9%). Consistent with previous reports, surgical resection showed a significant association with survival in both univariate and multivariate analyses. Better overall survival rate was related to surgical treatment closely (Fig. 5), and non-surgery performing was an independent risk factor for survival (Table 3). However, it was unable to distinguish the patients who had missed surgical indications from the patients who were unwilling to have an operation in SEER. Some situations regarding unable to perform surgery may produce an effect on survival. Even so, total removal of the tumor was an adequate treatment and the goal of the surgery by now was considered to decrease the local recurrence and improve the survival by avoiding re-operation [2]. Operative resection with wide resection margins could offer the best long-term prognosis [19], and inadequate resection margins had significantly worse oncologic outcomes [20]. We suggested that once patients with spinal chordoma were diagnosed at an early stage, surgical measures should be taken as far as possible. While spinal chordoma patients suffered metastasis in their initial diagnosis, further reasonable assessment for individuals according to life expectancy and therapeutic effect should not be ignored. In consideration of the low distant metastatic rates in chordoma, surgery was an effective and reliable treatment method for the majority.

The distant metastasis of spinal chordoma was rarely reported before. York et al. [21] showed that 5-year survival rate for lumbosacral chordoma patients is 86% compared with 40% for those in metastatic stage at presentation. Peter Bergh et al. [4] reported that surgical resection was not related to the rate of metastases. The metastatic rates of non-surgery spinal chordoma patients were almost the same as those of patients who underwent surgery. Our research revealed that only 7.7% cases of spinal chordoma presented with distant metastasis and the 5-year survival rate is 51.6%. Additionally, it was demonstrated that primary spinal chordoma patients with distant metastasis at presentation had a poorer prognosis than those with localized or regional cancer (Fig. 5).

The SEER database provided clinical information and survival outcome data based on a large group of people, but the study still had several limitations. Firstly, the SEER database was an unmatched resource in spinal chordoma with low incidence. Using death certificates to determine the cause of death could be inaccurate or difficult to interpret, so we chose to analyze overall survival instead of cause-specific survival. Secondly, we acknowledged that pelvic chordoma does not belong to spinal chordoma, but the primary tumor site code contained “sacrum” and “pelvis” together, so we could not screen out pelvic chordoma. Thirdly, we assumed that chordoma in the spine was more variable histologically, but we were unable to assess the impact of variant histological types on outcome because these were not recorded in the SEER database. Fourthly, age was reported as a categorical variable in 5-year intervals within the SEER program database, and an age of 20 years was arbitrarily chosen as a dividing standard of the study population. Finally, the SEER database only reported whether surgical intervention was performed, certain other variables, such as margin status, extent of surgical resection, and postoperative tumor recurrence, can not be analyzed retrospectively. Despite these shortcomings, the SEER program database served as an unparalleled resource when studying rare cancers.

## Conclusion

There was no significant difference in overall survival rate between chordomas located in the sacrum and vertebral column. Spinal chordoma patients with an elderly age (age ≥ 60), performed with non-surgical therapy, and with distant metastasis were associated with worse prognosis and poor overall survival. Performing surgery was an effective and reliable treatment method for patients, and public health efforts should pay more attention to elderly patients with spinal chordoma prior to distant metastasis.

## References

[1] Sundaresan N, Galicich JH, Chu FC, Huvos AG (1979). Spinal chordomas. J Neurosurg.

[2] Angelini A, Pala E, Calabrò T, Maraldi M, Ruggieri P (2015). Prognostic factors in surgical resection of sacral chordoma. J Surg Oncol.

[3] Lee IJ, Lee RJ, Fahim DK (2017). Prognostic factors and survival outcome in patients with chordoma in the United States: a population-based analysis. World Neurosurg..

[4] Bergh P, Kindblom LG, Gunterberg B, Remotti F, Ryd W, Meis-Kindblom JM (2000). Prognostic factors in chordoma of the sacrum and mobile spine: a study of 39 patients. Cancer.

[5] McMaster ML, Goldstein AM, Bromley CM, Ishibe N, Parry DM (2001). Chordoma: incidence and survival patterns in the United States, 1973–1995. Cancer Causes Control.

[6] Chen KW, Yang HL, Lu J, Liu JY, Chen XQ (2010). Prognostic factors of sacral chordoma after surgical therapy: a study of 36 patients. Spinal Cord.

[7] Jones PS, Aghi MK, Muzikansky A, Shih HA, Barker FG, Curry WT (2014). Outcomes and patterns of care in adult skull base chordomas from the Surveillance, Epidemiology, and End Results (SEER) database. J Clin Neurosci.

[8] Colli B, Al-Mefty O (2001). Chordomas of the craniocervical junction: follow-up review and prognostic factors. J Neurosurg.

[9] Boriani S, Bandiera S, Biagini R, Bacchini P, Boriani L, Cappuccio M (2006). Chordoma of the mobile spine: fifty years of experience. Spine (Phila Pa 1976).

[10] Surveillance, Epidemiology, and End Results Program: Overview of the SEER Program. http://seer.cancer.gov/about/overview.html. Accessed 20 Sept 2017.

[11] Baratti D, Gronchi A, Pennacchioli E, Lozza L, Colecchia M, Fiore M (2003). Chordoma: natural history and results in 28 patients treated at a single institution. Ann Surg Oncol.

[12] Mukherjee D, Chaichana KL, Gokaslan ZL, Aaronson O, Cheng JS, McGirt MJ (2011). Survival of patients with malignant primary osseous spinal neoplasms: results from the Surveillance, Epidemiology, and End Results (SEER) database from 1973 to 2003. J Neurosurg Spine.

[13] McGirt MJ, Gokaslan ZL, Chaichana KL (2011). Preoperative grading scale to predict survival in patients undergoing resection of malignant primary osseous spinal neoplasms. Spine J.

[14] Coffin CM, Swanson PE, Wick MR, Dehner LP (1993). Chordoma in childhood and adolescence. A clinicopathologic analysis of 12 cases. Arch Pathol Lab Med.

[15] Amendola L, Cappuccio M, De Iure F, Bandiera S, Gasbarrini A, Boriani S (2014). En bloc resections for primary spinal tumors in 20 years of experience: effectiveness and safety. Spine J.

[16] Hsieh PC, Xu R, Sciubba DM, MJ MG, Nelson C, Witham TF (2009). Long-term clinical outcomes following en bloc resections for sacral chordomas and chondrosarcomas: a series of twenty consecutive patients. Spine (Phila Pa 1976).

[17] Ahmed AR (2009). Safety margins in resection of sacral chordoma: analysis of 18 patients. Arch Orthop Trauma Surg.

[18] Guzik G (2015). Surgical treatment in patients with spinal tumors-differences in surgical strategies and malignancy-associated problems. An analysis of 474 patients. Ortop Traumatol Rehabil.

[19] Kayani B, Hanna SA, Sewell MD, Saifuddin A, Molloy S, Briggs TW (2014). A review of the surgical management of sacral chordoma. Eur J Surg Oncol.

[20] Kayani B, Sewell MD, Tan KA, Hanna SA, Williams R, Pollock R (2015). Prognostic factors in the operative management of sacral chordomas. World Neurosurg.

[21] York JE, Kaczaraj A, Abi-Said D, Fuller GN, Skibber JM, Janjan NA (1999). Sacral chordoma: 40-year experience at a major cancer center. Neurosurgery.

